# Editorial: Envisioning the Future of Industrial Bioprocesses Through Biorefinery

**DOI:** 10.3389/fbioe.2021.617999

**Published:** 2021-02-26

**Authors:** Jorge F. B. Pereira, Nicolas Papaiconomou, Sónia P. M. Ventura

**Affiliations:** ^1^Chemical Process Engineering and Forest Products Research Centre (CIEPQPF), Department of Chemical Engineering, University of Coimbra, Coimbra, Portugal; ^2^Institut de Chimie de Nice, UMR 7272, Université Côte d'Azur, CNRS, Nice, France; ^3^Aveiro Institute of Materials - CICECO, Chemistry Department, University of Aveiro, Aveiro, Portugal

**Keywords:** residues, biomass, circular economy, Biorefinery, downstream processes

Biorefinery is generally described as a facility integrating the conversion of biomass into new chemicals, fuels, and other commercial products and commodities. Supported by the Circular Economy concept, the Biorefinery scope is now enlarging to the conversion of residues, co-products and wastes. Worldwide, academia and industry believe that Biorefinery will soon play a crucial role in the economy, considering the outstanding growth of population and the increased needs for food, energy, chemicals, materials, pharmaceuticals, and other products, as well as acknowledging that society is moving toward a bio-based economy. For a successful market implementation of an integrated Biorefinery, all process unit operations must be technically improved. Moreover, the sustainability of the biomass-to-products chain must be guaranteed. This requires proposing new process developments ensuring both economically profitable bioprocesses and an absence of adverse environmental impacts.

Forestry, food, marine, and freshwater sectors are some of the most relevant players under the concept of Biorefinery, but they have been until now largely unexplored. Experts are working worldwide on the development of new or improved processes in order to address most issues attributed to so-called Biorefinery failure. Their efforts, however, are often not recognized and the resulting potential development of new products included in cosmetics, pharmaceutical, food, feed, medicine, and nutraceutical sectors are compromised. With this in mind, this Research Topic intends to call the attention of academia, industry and society for the chemical wealth that is yet being neglected, not only by the producers of these residues and raw materials, but also by the players of the principal sectors of activity worldwide. This Research Topic presents and discusses the relevant actions under development considering the theme of Biorefinery, particularly, through the analysis of the three main ivory towers of Biorefinery: (i) raw materials and residues; (ii) new or improved processes; and (iii) new products for old markets and old products for new markets. Contributions focused on microalgae, marine residues, and agro-food residues that are explored within a Biorefinery concept.

Agro-food residues and their biotechnological processing to obtain added-value products are central in industrial bioprocesses toward a more “ecofriendly” perspective, this considering the economic and environmental footprints. In this sense, this Research Topic started (Li et al.) by investigating the methane production by processing different parts of corn stover and using a simple co-culture of *Pecoramyces* and *Methanobrevibacter* species. The use of this simple co-culture of anaerobic and methanogen microorganisms allowed high methane conversion rates to be obtained from both the leaf blade and stem pith of corn stover, thus demonstrating its potential to convert lignocellulosic substrates into energy. The focus on other products than the production of bioenergy was reviewed by Mayolo-Deloisa et al.. In this work, the authors reported the valorization of by-products from the food industry (e.g., wastewater) as sources of laccase, a multi-copper oxidase that catalyzes the oxidation of a wide range of phenolic compounds. In this work, the authors focused on the enzyme bioprocessing, industrial potential and biotechnological applications in the food industry, calling the attention for the great potential of laccase to oxidize lignin, as a pretreatment of agro-food-wastes, greatly fitting the “reduce-reuse-recycle” strategy.

The valorization of freshwater biomass, particularly microalgae was also investigated in this Research Topic. Woortman et al. analyzed the total folate content and vitamer distribution in marine microalgae using stable isotope dilution assay (SIDA) accoupled to LC-MA/MS. High amounts of folate were detected in different microalgae, confirming the use of microalgae as a promising source of food-based compounds, as reported by Desai et al. In this second work focusing the valorization of microalgae, the feasibility of using aqueous solutions of ionic liquids based on imidazolium and phosphonium cations in order to separate hydrophilic and hydrophobic components from *Neochloris oleoabundans* was demonstrated. Results showed the capacity of ionic liquids to permeabilize and disrupt the microalgae cells, allowing the effective extraction of intracellular lipids, proteins and carbohydrates, under a multi-product scenario, and following the scope of a Biorefinery approach.

Interestingly, the use of ionic liquids was widely explored in this Research Topic. Shamshina and Berton scrutinized the recovery, dissolution, and treatment of chitin in ionic liquids. In this review, recent developments in the processing of chitin, particularly using ionic liquid-based techniques (as solvents, co-solvents, or catalysts) were highlighted, demonstrating how this type of solvents improves the chitin processing. The importance of valorization of biomass in a circular economy and bioeconomy precepts was also underlined by both authors, specially, emphasizing the “paradigm shift” needed to balance the oil- to biopolymer-based chemicals, such as chitin for example. The positive impact of ionic liquids in biotechnological processes was also the focus of the original work of Dinis et al. Here, the authors investigated the use of aqueous solutions of ionic liquids as potential media on the DNA stability. The main results have confirmed pH as the most significant condition impacting the DNA stability, and cholinium-based ionic liquids as the most promising preservation agents. While in this work, the authors studied the DNA stability, this investigation also shed light on the interesting potential of using ionic liquids as preservation agents and stabilizing solvents for other molecules of interest, namely proteins and enzymes extracted from different biomasses (e.g., micro and macroalgae). The seventh work composing this Research Topic looked to another perspective of biotechnology industry, though strongly connected to the potential valorization of bioactive products of freshwater and marine origin. In this specific work, the authors developed a purification process able to obtain antigens for vaccine production. After a careful optimization, the authors were able to improve the purification of these antigens (pneumococcal capsular polysaccharides) by integrating an ultrafiltration with precipitation, demonstrating a higher immunogenicity of the conjugate vaccine prepared. In the end, the quality of the results, protocols, and reviews compiled in this Research Topic have caught our interest, and we hope that these will stimulate all scientific communities related to Biotechnology to move forward the investigation based on sustainability and circularity concepts associated with the industrial biorefineries.

## Author Contributions

SV, JP, and NP have contributed equally for this Editorial. All authors contributed to the article and approved the submitted version.

## Conflict of Interest

The authors declare that the research was conducted in the absence of any commercial or financial relationships that could be construed as a potential conflict of interest.

